# Metabolomics Workbench: An international repository for metabolomics data and metadata, metabolite standards, protocols, tutorials and training, and analysis tools

**DOI:** 10.1093/nar/gkv1042

**Published:** 2015-10-13

**Authors:** Manish Sud, Eoin Fahy, Dawn Cotter, Kenan Azam, Ilango Vadivelu, Charles Burant, Arthur Edison, Oliver Fiehn, Richard Higashi, K. Sreekumaran Nair, Susan Sumner, Shankar Subramaniam

**Affiliations:** 1 San Diego Supercomputer Center, University of California, San Diego, 9500 Gilman Drive, La Jolla, CA 92037, USA; 2 University of Michigan, 6300 Brehm Tower, 1000 Wall Street, Ann Arbor, MI 48105, USA; 3 University of Florida, 2250 Shealy Drive, Gainesville, FL 32608, USA; 4 University of California, Davis, 451 Health Sciences Dr, Davis, CA 95616, USA; 5 University of Kentucky, 789 S. Limestone, 521 Biopharm Bldg, Lexington, KY 40536, USA; 6 Mayo Clinic, 200 First street SW, Rochester, MN 55902, USA; 7 RTI International, 3040 Cornwallis Rd, Research Triangle Park, NC 27709, USA; 8 Departments of Bioengineering, Computer Science and Engineering, Cellular and Molecular Medicine, and Chemistry and Biochemistry, University of California, San Diego, La Jolla, CA 92093, USA

## Abstract

The Metabolomics Workbench, available at www.metabolomicsworkbench.org, is a public repository for metabolomics metadata and experimental data spanning various species and experimental platforms, metabolite standards, metabolite structures, protocols, tutorials, and training material and other educational resources. It provides a computational platform to integrate, analyze, track, deposit and disseminate large volumes of heterogeneous data from a wide variety of metabolomics studies including mass spectrometry (MS) and nuclear magnetic resonance spectrometry (NMR) data spanning over 20 different species covering all the major taxonomic categories including humans and other mammals, plants, insects, invertebrates and microorganisms. Additionally, a number of protocols are provided for a range of metabolite classes, sample types, and both MS and NMR-based studies, along with a metabolite structure database. The metabolites characterized in the studies available on the Metabolomics Workbench are linked to chemical structures in the metabolite structure database to facilitate comparative analysis across studies. The Metabolomics Workbench, part of the data coordinating effort of the National Institute of Health (NIH) Common Fund's Metabolomics Program, provides data from the Common Fund's Metabolomics Resource Cores, metabolite standards, and analysis tools to the wider metabolomics community and seeks data depositions from metabolomics researchers across the world.

## INTRODUCTION

The small molecule metabolites are involved in various physiological processes, and play an important role in the regulation and control of cellular function and disease. It is vital to systematically identify, quantify, and characterize these metabolites across various cellular processes, in order to understand their involvement in these processes. These efforts broadly fall under the field of metabolomics (1–6). Metabolomics studies result in the measurement of metabolites, which are end-point readouts of biological processes/pathways often measurable in the serum, in contrast to data from genomics, transcriptomics and proteomics studies. It has now become routine to measure the metabolites through various combinations of MS and NMR techniques coupled with liquid chromatography (LC) and gas chromatography (GC) methods. The National Institute of Health (NIH) Common Fund's Metabolomics Program (https://commonfund.nih.gov/metabolomics/index), a multi-institutional and multi-year program, is spearheading an effort to accelerate the development of the metabolomics field by establishing programs in the following areas: creation of metabolomics resource cores to conduct comprehensive metabolomics studies; establishment of training programs; development of new metabolomics technologies to address limitations of current technologies; increasing the number of chemically identifiable metabolites through the synthesis and availability of high quality reference standards; promoting data sharing and international collaboration.

The Data Repository and Coordinating Center (DRCC), as part of the NIH Common Fund's Metabolomics Program, has developed the Metabolomics Workbench to promote data sharing and collaboration. The Metabolomics Workbench provides a computational platform to integrate, analyze, track, deposit, and disseminate large volumes of data from metabolomics studies performed by the NIH-funded Regional Comprehensive Metabolomics Resource Cores (RCMRCs) and their collaborators, and other metabolomics researchers, along with access to protocols and metabolite standards for metabolomics studies. A wide variety of statistical analysis tools have also been developed and integrated into the Metabolomics Workbench for exploratory comparative analysis of experimental measurements data within studies available on the Workbench.

Examples of existing metabolomics data repositories and databases include MetaboLights (7) (www.ebi.ac.uk/metabolights), MassBank (8) (www.massbank.jp), LIPID MAPS (9) (www.lipidmaps.org), BioMagResBank (10) (www.bmrb.wisc.edu), Human Metabolome Database (HMDB) (11) (www.hmdb.ca), METLIN (12) (http://metlin.scripps.edu), KNApSACK (http://kanaya.aist-nara.ac.jp/KNApSAcK/), ChemSpider (13) (www.chemspider.com) and MetaCyc (14) (www.metacyc.org). The support for public data deposition along with its dissemination to the research community is not available across all these resources. An integrated set of tools for exploratory analysis is generally not present in most of the existing data repositories. The Metabolomics Workbench serves as a public data repository for metabolomics metadata and experimental data, and a portal for metabolite standards, metabolite structures, protocols, tutorials and training material, and other educational resources, along with integrated set of exploratory analysis tools. We describe our current and ongoing work on the Metabolomics Workbench in the following sections.

## DATABASE CONTENT AND DESCRIPTION

### Metadata and experimental data

**Figure 1.**
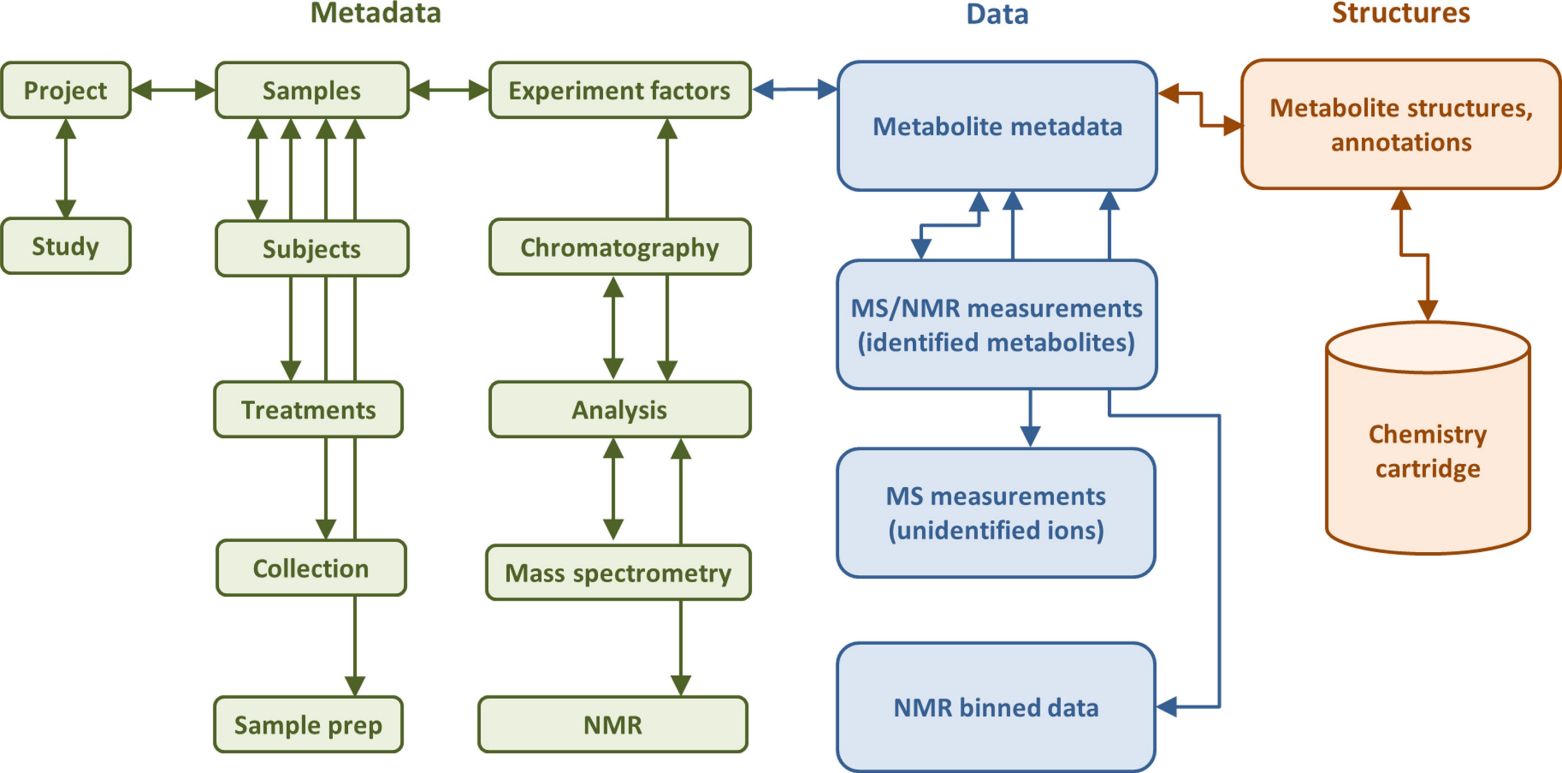
An overview of data captured for studies in the Metabolomics Workbench.

The availability of metadata along with experimental measurements data for the metabolomics studies is important not only for defining experimental methodology but also for facilitating reproducibility and analysis of studies by the wider community of metabolomics researchers. To that end, the Metabolomics Workbench requires that metadata must be provided, in addition to experimental raw data and processed measurements data, during the deposition of studies to the Workbench. A substantial body of literature exists regarding the requirements for collecting metadata for metabolomics studies involving humans, plants and environmental studies. The Metabolomics Standards Initiative (MSI) (15), in particular, has developed recommendations for minimum metadata reporting standards for performing metabolomics studies and analyzing data for biological samples in mammalians/*in vivo* and microbial/*in vitro* experiments, plant biology, MS and NMR experiments, and chemical analysis (16–21). The Metabolomics Workbench has adopted recommendations compiled by MSI and has developed a Microsoft (MS) Excel-based metadata template containing appropriate metadata fields to cover a wide variety of metabolomics studies. The required metadata fields in the metadata template file must be filled in by the user submitting the data and uploaded to the Workbench along with experimental raw and processed data. Figure 1 shows an overview of metadata and experimental data being captured for studies submitted to the Workbench.

The metadata template consists of the following key sections to cover metadata reporting standards recommended by MSI: project, study, experimental design, subjects, treatment, collection, sample preparation, chromatography, analysis and MS/NMR. Each metadata section in turn contains a set of required and optional data fields pertaining to appropriate details about the experiment. For example, the analytical metadata sections include details regarding sample storage conditions, sample preparation and extraction protocols, sample procurement and analytical methods.

In addition to the metadata component, the Workbench data repository collects the corresponding experimental raw and processed measurements data. Processed experimental metabolite measurements may be in the form of quantitated metabolites concentrations, MS peak height or area, LC retention times and NMR binned areas. Raw experimental data in the form of binary files along with any associated instrumental and experimental parameters may also be deposited into the Workbench. The depositing data subsection, under the database implementation and user interfaces section, provides additional details regarding file formats for uploading processed and raw experimental data to the workbench.

Table 1 provides an overview of metadata and experimental data currently available in the Metabolomics Workbench.

**Table 1.** An overview of the number of metadata and experimental data entries in the Metabolomics Workbench as of August 2015

Total studies	215	Species	25	Untargeted MS data m/z values	305 505
Public studies	145	Human studies	96	Protocols	135
Embargoed studies	70	MS studies	170	Standards (synthesis completed, in process)	9, 22
Projects	145	NMR studies	47	Raw studies data for download	926G
Institutes	53	Measured metabolites	59 754		

### Metabolites structure data

The Workbench metabolite structure database contains approximately 60 000 curated chemical structures and annotations of biologically relevant metabolites. The metabolite structures were collected mainly from public repositories such as LIPID MAPS (22) (www.lipidmaps.org), ChEBI (23) (www.ebi.ac.uk/chebi), HMDB (11) (www.hmdb.ca), BMRB (10) (www.bmrb.wisc.edu), PubChem (24) (http://pubchem.ncbi.nlm.nih.gov) and KEGG (25) (www.genome.jp/kegg/). Additionally, the metabolite structure database contains structures of metabolites identified by the data depositors in their metabolomics experiments.

In addition to curation of metabolite structures and assignment of common properties such as name, systematic name, exact mass and molecular formula, the metabolites are classified either using the LIPID MAPS classification systems for lipids (26) or by the ClassyFire classification system (Feunang, Y.D. and Wishart, D.S. Unpublished work), a tool for automated structure-based taxonomic classification of chemical entities.

Each metabolite in the structure database is also mapped to the following external database IDs, to the extent possible: PubChem CID, LMSD ID, CHEBI ID, HMDB ID, Chemspider ID, METLIN ID, BMRB ID and MetaCyc ID. A key set of the following physicochemical properties are calculated for all metabolites and loaded into the database: number of heavy atoms, number of rings and aromatic rings, number of rotatable bonds, van der Waals molecular volume, topological polar surface area, number of hydrogen bond donors and acceptors, logP, molar refractivity, fraction sp3 carbons and number of sp3 carbon. The physicochemical properties of metabolites in the database are calculated using an external open source package named MayaChemTools (www.mayachemtools.org).

### Metabolomics protocols and standards

The protocols associated with the studies deposited in the Workbench or provided as general purpose protocols by the RCMRCs are maintained as a separate collection of documents in the workbench. Theses protocols are annotated using keywords such as analysis type (MS/NMR), metabolite class, sample type and process. The protocols involved in any studies are also linked to those studies.

The Metabolite Standards Synthesis Cores, as part of the NIH Common Fund's Metabolomics Program, perform synthesis of high quality metabolite standards, in order to make them available to a wider community of metabolomics researchers. The compounds nominated for synthesis are reviewed by the executive committee of the NIH Common Fund and prioritized for synthesis. After the compounds have been synthesized, they are made available to the metabolomics researchers through the Workbench. Any researcher may request an aliquot. Currently, the synthesis of 9 compounds has been completed; synthesis is in process for an additional 22 compounds.

### Tutorials, training material and other resources

An important goal of the NIH Common Fund's Metabolomics Program is to provide training and mentoring opportunities to a broad spectrum of researchers interested in metabolomics. To that end, the Workbench maintains and provides an extensive list of events and training resources, including tutorials and videos of interest to the metabolomics community. The material available on the Workbench covers a wide variety of topics, and may be of interest to researchers ranging from those who are starting in the field of metabolomics to metabolomics experts who may be interested in learning about recent advances in metabolomics research and technologies.

The Workbench also maintains links to events, instructions, and hands-on training materials generated by the RCMRCs and their collaborators spanning the following topics of interest to the metabolomics community: practice and theory in metabolomic applications, metabolomics investigative process, state of metabolomics technologies, hands-on training in MS/NMR spectrometry, stable isotope-resolved metabolomics, data processing, data interpretation, tools for data visualization, etc. A set of instructional video series with videos covering various aspects of metabolomics practices is also available.

## DATABASE IMPLEMENTATION AND USER INTERFACES

**Figure 2.**
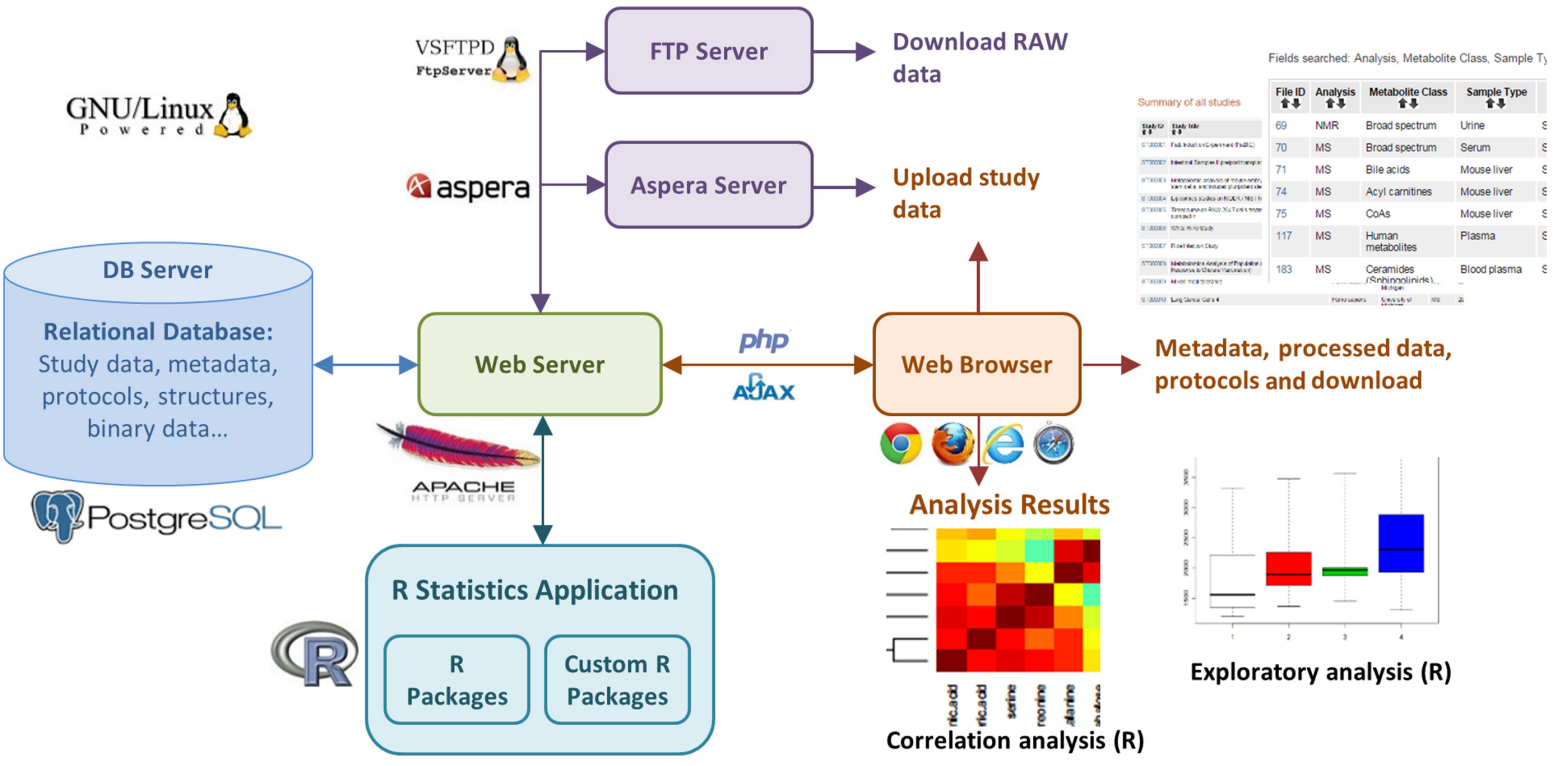
An overview of the Metabolomics Workbench systems architecture.

The Metabolomics Workbench metadata, experimental measurements data, and metabolites structure data along with other associated data are stored in an open source PostgreSQL Version 9.0.13 (www.postgresql.org) relational database deployed on a virtual Linux machine running under GNU/Linux operating system Version 2.6.18. PHP and Perl scripts are used to validate, parse and load data into the database. The chemical structure data in MDL MOL file format (http://accelrys.com/products/collaborative-science/biovia-draw/ctfile-no-fee.html) is stored in a database table as a text string. An open source Bingo Chemical Cartridge (http://lifescience.opensource.epam.com/bingo/index.html) is deployed to support substructure and similarity searching across metabolites structures. The Workbench browse/query graphical user interfaces (GUI) and presentations of search results are implemented through a combination of PHP and Perl scripts using the Apache HTTP server Version 2.2.3 running on a virtual Linux machine. An open source secure FTP server, VSFTP Version 2.0.5, is deployed on the workbench to support anonymous download of all available data for the studies in the workbench through standard web browsers or standalone command line file transfer clients. Data files are uploaded to the workbench using Aspera Connect (http://asperasoft.com/software/transfer-clients/connect-web-browser-plug-in), a self-installing web browser plug-in that powers high-speed uploads through Aspera Connect Server running on the workbench server, which is capable of supporting upload speeds up to 100 Mbps. Figure 2 provides the overview of systems architecture for the Metabolomics Workbench.

### Depositing data

The ‘Upload and Manage Data’ page under the ‘Data’ tab on the Metabolomics Workbench enables users to upload metadata and experimental measurements data, along with raw experimental data, for deposition into the Workbench. The ability to list already deposited data sets is available. User registration is required for data deposition to the Workbench.

The metadata and experimentally measured results data must be submitted to the Workbench either by filling in the MS Excel metadata and results templates available on the Workbench (www.metabolomicsworkbench.org/data/DataTemplates/DRCCMetadataAndResultsTemplates.zip) or as an mwTab text file. The metadata template is designed to cover metadata reporting standards recommended by MSI. The results template captures details about experimental data such as metabolite names, sample IDs and measured values. Alternatively, the metadata and experimentally measured data may be submitted to the workbench as an mwTab text file, a format developed by the Metabolomics Workbench, in order to facilitate programmatic processing of data suitable for submission to the Workbench.

The metadata, experimentally measured data, and raw experimental data are submitted to the Workbench as a single compressed file. The raw experimental data files may consist of any type of relevant binary or text data files including instrument files. After the metadata and experimental data have been deposited in the workbench, a series of data validation, curation and processing steps, employing a combination of both manual and automatic data processing strategies, are performed followed by an assignment of a study identifier to the deposited data. The processed metadata and experimental data are made available to the user for further review at a secure private website. After the user has reviewed the processed data for a study and signed off on its release to the public, the study is made available on the public Workbench server, subject to any requested embargo date.

### Browsing/searching experimental studies

The Metabolomics Workbench ‘Browse/Search’ page under the ‘Data’ tab provides the capability to view metadata along with associated experimentally measured data either by projects or studies. After the user selects one of these two options, a corresponding summary page for projects or studies is presented. The project summary page contains information such as project titles, name of the institutes where experiments were performed and analyzed, and the number of studies present in each project. The project ID on the projects summary page is hyperlinked to the project detail page, which displays relevant metadata for the project along with a tabular summary of all the studies in that project. The study summary page lists study IDs along with the following information for each study in a tabular format: study title, species, institute name, type of analysis, data submission date, number of samples and a link to download all available study data.

Selection of a study ID from either the projects or studies summary page brings up a study-specific detail page providing access to all metadata and experimental data for the selected study. All available metadata information about the study such as study ID, title, study type, institute, number of study groups, total subjects and investigator details, is summarized at the top of the page. After the study summary information section, the following tabs are provided to allow the user to access all available metadata details for the study: all, project, study design, treatment, collection, sample preparation, chromatography, analysis and MS or NMR. Additional links are available at the top of the study detail page to allow the user to view metadata for study samples, experimentally measured data on named metabolites, NMR binned data, and view/download all targeted and untargeted experimental data. The user can view all available experimental measurement data for all samples in a study or generate variety of plots to perform exploratory analysis on a set of selected experimental measurements for the study.

The Workbench metadata and experimental data query page enables the user to search the Workbench database by any combination of the following data fields: subject type, species, project or study title, experimental or analysis institute, year submitted and analysis type. Selection of MS or NMR from analysis type drop-down menu causes additional MS (type, ion mode, instrument type) or NMR (experiment type, instrument type, spectrometer frequency) search parameters to appear below the analysis type. The results of the search may be displayed by either projects or studies. Initiating a search using the default page, without specifying any specific search parameters, retrieves all available data from the database and displays the results as project summary pages.

**Figure 3.**
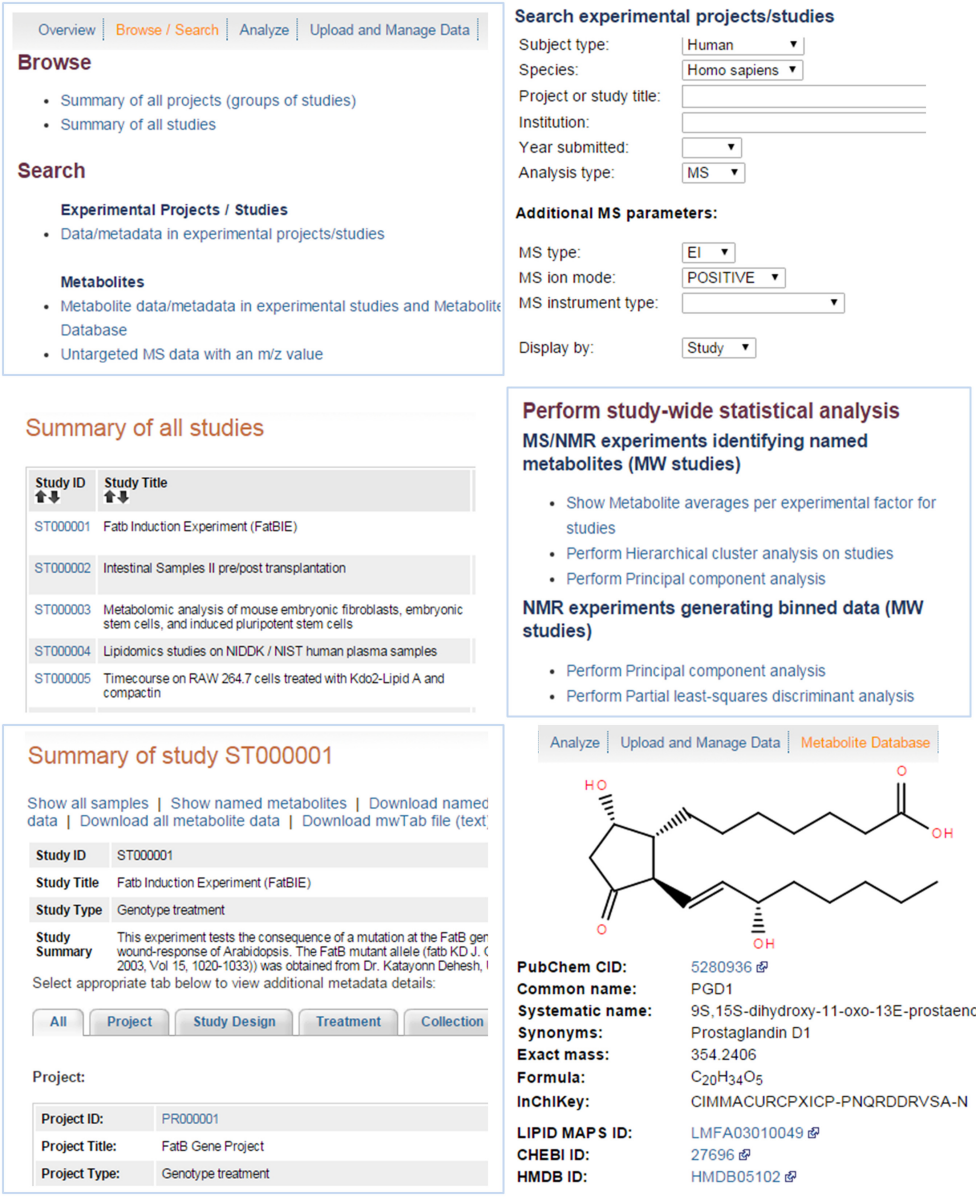
A montage of the Metabolomics Workbench screen shots showing different ways to search, view, retrieve and analyze metabolomics data.

Figure 3 shows screen shots of user interfaces for the Metabolomics Workbench depicting different ways to search, retrieve, view, and analyze metabolomics metadata and experimentally measured data along with a screen shot depicting details of a metabolite structure database record.

### Searching the metabolites structure database

The Workbench provides three different ways to search the metabolites structure database: a substructure-based search, a text-based search and a mass (m/z) search. The structure-based search page allows the user to search the metabolites structure database by performing a substructure, exact, or Tanimoto structure match using the structure drawn in Ketcher (http://lifescience.opensource.epam.com/ketcher/index.html), an open source JavaScript-based structure drawing package. The user can also search the metabolites structure database by any combination of the following database fields using the text-based search page: PubChem CID, name (common, systematic or synonym), chemical formula and exact mass.

The results summary page for both the structure- and text-based searches displays a table containing all metabolites from the metabolites database matching specified search criteria. The result table contains the following data columns: metabolite structure ID, PubChem CID, name, systematic name, formula and exact mass. The user may select the structure ID of any metabolite from the results page and display the structure of the selected metabolite along with additional information such as common and systematic name, synonyms, exact mass, formula, InChiKey, chemical classification, calculated physiochemical properties, and various external database IDs such as LIPID MAPS ID, HMDB ID, KEGG ID and METLIN ID.

The ‘MS search’ option under the ‘Data’ tab on the Metabolomics Workbench provides the capability to perform precursor-ion MS searches against the metabolite database, a computationally generated database of approximately 30 major lipid classes, and a curated subset of the metabolite database composed of approximately 6000 reference metabolites which can be routinely detected by MS. The user may input peaklists directly in the search form, upload a peaklist file, or alternatively input all unidentified MS precursor-ions from a particular study of interest. The user may set parameters such as ion type and mass tolerance range, and also limit the search to particular classes of metabolites. Depending on the context and the choice of target database, the search results page shows the results in a tabular format containing the metabolite, name, input m/z, exact m/z, delta corresponding to the absolute difference between the experimental and the calculated m/z value for a particular ion mass, formula, exact mass and ion type. Additionally, links to structures in the metabolite database or examples of generic structures corresponding to computationally generated structures in the metabolite structure database are also shown. The MS search results are also integrated with metabolite information in the experimental studies, in order to provide access to all studies containing experimentally measured metabolites which match results of an MS search.

### Analyzing data

The ‘Analyze’ page provides access to the following exploratory statistical analysis methods, in order to compare and analyze experimental data in studies available on the Workbench: calculation of metabolite averages per experimental factor; hierarchical cluster analysis; principal component analysis; partial least-squares discriminant analysis; linear discriminant analysis; clustered correlation analysis; network analysis on correlated metabolites; volcano analysis on pairs of experimental factors; relative log abundance plots. These statistical analysis tools are implemented in the R statistical package (www.r-project.org) using custom R code and various open source R modules such as hclust, prcomp, muma (27), mixOmics (www.mixomics.org), metabolomics and VolcanoPlot.

The user can perform statistical analysis of experimentally measured data corresponding to named metabolites in MS/NMR studies or binned data in NMR studies. The analysis tools are integrated into the Workbench to allow the user to analyze all experimental data for named metabolites in all available studies, without the need to first generate a formatted data file. The users may also download the results for studies in the Workbench as tab-delimited text files for analysis by their own analysis tools or any other external analysis tools. After the user selects a specific analysis type to perform on MS/NMR experimental data, an analysis summary page displays a tabular view of only those studies which contain experimental data appropriate for the selected analysis. On completion of a specific analysis for the selected study, the numerical and graphical results are loaded into the browser, which allow the user to download and save the results for further analysis.

### Downloading data

The Metabolomics Workbench provides multiple ways to download data for publicly released studies. An anonymous FTP server (ftp://www.metabolomicsworkbench.org) is available to download data for individual studies as compressed ZIP files containing all the data uploaded by the user, including any raw data files along with metadata in MS Excel files. The user can simply click on a ZIP file to initiate its download in the browser. Alternatively, a variety of free and commercial standalone file transfer clients exist to download large ZIP files directly from the anonymous FTP server.

Additionally, the Workbench provides the capability to retrieve data directly from the database using REpresentational State Transfer (REST) (www.ics.uci.edu/∼fielding/pubs/dissertation/rest_arch_style.htm) services via HTTP requests. The REST service URL may be used to retrieve data in a browser or embedded in command line programs or scripts to allow programmatic access. The Workbench REST service URL consists of three parts: the invariant base URL, the input specification and the output specification. Further details about the format of these specifications, along with various examples to retrieve data from the database, are provided on the Workbench (www.metabolomicsworkbench.org/data/mw_rest.php). An interactive REST URL creator is also available on the Workbench to facilitate rapid generation of the URL needed to retrieve data of interest from the Workbench database.

## SUMMARY AND FUTURE WORK

The Metabolomics Workbench is a public repository for metabolomics metadata and experimental data spanning various species and experimental platforms, metabolite standards, metabolite structures, protocols, tutorials and training material and other educational resources. It provides a computational platform to integrate, analyze, track, deposit and disseminate large volumes of heterogeneous data from a variety of metabolomics studies.

We are currently collaborating with the MetaboLights project team at the European Bioinformatics Institute (EBI) to explore and implement ways to facilitate metabolomics data exchange among different data repositories. To that end, an online resource called metabolomeXchange (http://metabolomexchange.org/) has been set up listing metadata sets available in the following metabolomics data repositories: Golm Metabolome Database, Metabolomic Repository Bordeaux, MetaboLights and Metabolomics Workbench. Initial discussions are underway to develop flat file data exchange formats to allow automatic sharing and processing of complete data sets between MetaboLights and Metabolomics Workbench data repositories.

In addition to maintenance and processing of new data sets deposited in the Workbench, we anticipate development of additional data processing, analysis, and visualization tools as needed to accommodate new types of data. We plan to make these tools available to the wider metabolomics community through the Workbench.
